# Intestinal parasitosis, anaemia and risk factors among pre-school children in Tigray region, northern Ethiopia

**DOI:** 10.1186/s12879-020-05101-8

**Published:** 2020-05-27

**Authors:** Araya Gebreyesus Wasihun, Mekonen Teferi, Letemichal Negash, Javier Marugán, Dejen Yemane, Kevin G. McGuigan, Ronan M. Conroy, Haftu Temesgen Abebe, Tsehaye Asmelash Dejene

**Affiliations:** 1 Department of Medical Microbiology and Immunology, College of Health Sciences, Mekelle University, Tigray, Ethiopia; 2 Department of Biology, College of Natural and Computational Sciences, Mekelle University, Tigray, Ethiopia; 3 Department of Chemical and Environmental Technology, Universidad Rey Juan Carlos, C/ Tulipán s/n, 28933 Móstoles, Madrid, Spain; 4 Department of Environmental Health, College of Health Sciences, School of Public Health, Mekelle University, Tigray, Ethiopia; 5 Department of Physiology and Medical Physics, Royal College of Surgeons in Ireland (RCSI), Dublin 2, Ireland; 6 Data Science Centre, Royal College of Surgeons in Ireland (RCSI), Dublin 2, Ireland; 7 Department of Bio Statistics, College of Health Sciences, School of Public Health, Mekelle University, Tigray, Ethiopia; 8 Department of Medical Microbiology, College of Health Sciences, Aksum University, Tigray, Ethiopia

**Keywords:** Intestinal parasitic infections, Anaemia, Pre-school age children, Risk factors, Ethiopia

## Abstract

**Background:**

Intestinal parasitic infections (IPIs) and anaemia are major health problems. This study assessed the prevalence of intestinal parasitic infections, anaemia and associated factors among pre-school children in rural areas of the Tigray region, northern Ethiopia.

**Methods:**

A community based cross-sectional study was conducted among 610 pre-school children in rural communities of Northern Ethiopia from June 2017 to August 2017. Stool specimens were examined for the presence of trophozoites, cysts, oocysts, and ova using direct, formal-ethyl acetate concentration, Kato–Katz, and Ziehl-Neelsen techniques. Haemoglobin was measured using a HemoCue spectrometer.

**Results:**

Among the 610 participating pre-school children in the study, the prevalence of IPIs and anaemia were 58% (95% conference interval (CI): 54.1–61.9%) and 21.6% (95% CI: 18.5–25.1%), respectively. Single, double, and triple parasitic infections were seen in 249 (41, 95% CI: 37–45%), 83 (14, 95% CI: 11–17%), and 22 (3.6, 95% CI: 2.4–5.4%) children, respectively. Of the seven intestinal parasitic organisms recorded from the participants, *Entamoeba histolytica/dispar* was the most prevalent 220 (36.1%) followed by *Giardia lamblia* 128 (20.1%), and *Hymenolepis nana* 102 (16.7%). Mixed infections were common among *G. lamblia, E. histolytica/dispar* and *Cryptosporidium* spp. oocyst. Intestinal parasitic infection prevalence increased from 47% in children aged 6–11 months to 66% in those aged 48–59 months; the prevalence ratio (PR) associated with a one-year increase in age was 1.08 (95% CI: 1.02–1.14, *p* = 0.009). Age-adjusted prevalence was higher in children who had been dewormed (PR = 1.2; 95% CI: 1.00–1.4, *p* = 0.045), and lower in households having two or more children aged under five (PR = 0.76, 95% CI: 0.61–0.95, *p* = 0.015). Anaemia rose from 28% in children aged 6–11 months to 43% in those aged 12–23 months, then fell continuously with age, reaching 7% in those aged 48–59 months. Age adjusted, anaemia was more prevalent in households using proper disposal of solid waste (PR = 1.5, 95% CI: 0.1–2.10, *p* = 0.009) while eating raw meat (PR = 0.49, 95% CI: 0.45–0.54, *p* = 0.000), any maternal education (PR = 0.64 95% CI: 0.52–0.79, p = 0.000), and household water treatment (PR = 0.75, 95% CI: 0.56–1.0, *p* = 0.044) were associated with lower prevalence of anaemia.

**Conclusions:**

More than half of the children were infected with intestinal parasites, while anaemia prevalence was concentrated in the 12–23 month age group. This study has identified a number of potentially modifiable risk factors to address the significant prevalence of IPIs and anaemia in these children. Improvements in sanitation, clean water, hand hygiene, maternal education could address both short and long-term consequences of these conditions in this vulnerable population.

## Background

Intestinal parasitic infections (IPIs) are an important cause of morbidity and mortality worldwide [1] affecting about 3.5 billion people globally [2]. IPIs are endemic in resource-limited regions due to high population density, low access to improved water sources, low latrine availability, poor hygiene conditions, low health awareness, and limited economic resources [3, 4]. Helminths such as *Ascaris lumbricoides,* Hookworm*, Strongloides stercolaris* and *Trichuris trichiura,* and enteric protozoan parasites such as *Entamoeba histolytica, Giardia lamblia* and *Cryptosporidium* spp. cause high incidences of health problems especially in children in low to middle income countries [5].

Children due to their immature immune systems and frequent exposure to unhygienic environments are at high risk for IPI including helminths [6] and protozoa [7]. These infections are common during the period of life most critical for physical and cognitive development, hence are linked with an increased risk of childhood malnutrition and growth deficits [8]. Poor health in children also results in deficits in cognitive development and educational achievements [9].

As with IPIs, anaemia remains a public health problem affecting both developing and developed countries with major consequences for human health as well as social and economic development [10]. Globally, 2011 data indicate that 43% of children under-five were anaemic, with a higher prevalence in the developing world, specifically South Asia and East Africa, being 58 and 55%, respectively [11]. Sub-Saharan Africa is one of the most affected regions with 54% of children under-five suffering from anaemia [12]. The causes of anaemia include folate and iron deficiencies [13], malaria [14], infections (e.g., intestinal helminths), and diarrhoea [15]. Childhood anaemia has many irreversible impacts: it impairs physical growth [16], impairs immune function*,* increases susceptibility to infections [17] and weakens motor development leading to reduced cognitive ability [18] and short or long term mortality in severe cases [13].

The 2016 Demographic and Health Survey of Ethiopia (EDHS) report showed that the national prevalence of anaemia among children aged 6 to 59 months was 57% [19], which exceeds the 40% threshold set by the World Health Organization (WHO) [13]. The EDHS report of anaemia prevalence in the Tigray regional state (54%) is marginally below the national average (57%) [19].

Most studies conducted in Ethiopia on the prevalence of IPIs are on school-age children. Studies conducted among pre-school age children have shown IPI prevalence up to 85.1% [20]. Similarly, national studies on anaemia prevalence report prevalence from 32 to 37% [21]. These studies, however, are focused on either soil transmitted helminths alone [22] or symptomatic hospitalized children [23], or investigated anaemia alone. Furthermore, none of the above studies used modified Ziehl-Neelsen techniques to detect *Cryptosporidium* spp., the second most causative agent of diarrhea among children under five next to rotavirus [24].

There is scarcity of data on the prevalence of IPI, anaemia, and their associated risk factors among pre-school children in the study area. However, the prevalence is expected to be high given the poverty, poor hygiene, hot/humid tropical climate and lack of access to potable water. Establishing baseline prevalence and elucidating potentially modifiable risk factors for IPI and anaemia would help public health planners, policy makers and implementers to plan and design appropriate intervention strategies to reduce associated morbidity and mortality among pre-school children.

## Methods

### Study area, design, study population, setting and period

This community based cross-sectional study was carried out from June–August 2017 in rural communities surrounding the Mekelle zone, Tigray region, northern Ethiopia. Four sites namely: Serawat, Harena, Maynebri and Tsuwanet were selected using a simple random lottery method from the total of 32 administrative unit found in the surrounding districts. The population typically experience poor sanitation, poor access to safe drinking water, and low socioeconomic status. More details have been previously described elsewhere [25].

### Sample size and sampling technique

The sample size of the study was determined using a single population proportion formula, considering an estimate of 24.3% expected prevalence of IPIs among children younger than 5 years old. Assuming any particular outcome to be within a 5% marginal error and a 95% confidence interval of certainty, the final sample size with a design effect of two is 570 (based on Statulator website, http://statulator.com/SampleSize/ss1P.html). To allow for data loss, 610 participants were recruited.

We used a multistage stratified sampling technique to identify study participants after the kebelles (a kebelle is the smallest local government administrative unit in Ethiopia) were stratified. In the selected kebelles, 2674 children aged 6–59 months were identified with their respective households using the registration at health posts and through the health extension workers.

We allocated the calculated sample proportionally to the selected kebelles based on the total number of households with children aged 6–59 months in each kebelle. Study participants were then identified using simple random sampling of the households. In cases where households had more than one eligible child, the eldest child was included. Accordingly, the distribution of households with respect to the kebelles was, 133 from Tsawnet, 142 from Harena, 158 from Serawat, and 177 from Mynebri**.**

### Data collection

After written consent was obtained from mothers or guardians of eligible children, socioeconomic, environmental, behavioural and health related data were collected using a structured questionnaire (translated from English and printed in the local Tigrigna language). Data were collected using a face-to-face administrated questionnaire and an observation method by trained data collectors, under the supervision of the principal investigators. Child hand cleanness and nail status in addition to toilet availability were assessed by direct observation.

### Out come variables

IPIs and anaemia among children aged 6–59 months (pre-school children).

### Predictor variables

#### Socio-economic variables

Gender and age of the study child, mother’s/guardian’s educational status and occupation, family size, family income, number of children 6–59 months in the household.

#### Environmental and behavioral variables

Consumption of raw vegetables, child contact with pet animals, child deworming, habit of playing in soil, shoe wearing habit, child hand cleanliness and fingernail status. Use of soap for hand washing, water source, use of household water treatment, latrine availability, and type of drinking water source.

#### Faecal sample collection

Following the completion of consent and questionnaire, a clean, wide screw-capped plastic stool cup, labelled with names was provided to each mother/guardian. They were requested to bring about 10 g (thumb size) fresh stool from their child to the nearby health posts the following day within 10–30 min of passage. Participants who were not able to provide a sample on the first day were asked again on the following day.

#### Haemoglobin (Hb) measurement

Finger-prick blood specimens were obtained from participants to assess Hb levels using a HemoCue analyser in the health post (HemoCue Hb 201z, Sweden) (49). The apparatus was calibrated daily using the reference micro cuvettes as indicated by the manufacturer. Definition and classification of anaemia were according to the WHO cut-offs [26].

### Parasitological analysis

Stool specimens were analyzed at the respective health posts by three trained laboratory technicians using direct saline wet mount, formalin ethyl acetate concentration technique [27] and single Kato–Katz technique (thick smear 41.7 mg). For the detection of *Cryptosporidium* spp. oocysts, modified Ziehl-Neelsen (MZN) staining was performed. Kato–Katz, wet mount preparations and modified Ziehl-Neelsen were analysed within 1 h of preparation in each respective health posts to detect hookworm eggs, protozoa parasites (*E. histolytica/dispar* and *G. lamblia*), and *Cryptosporidium* spp., respectively.

The remaining stool specimens were transported in screw-capped cups in 10% formalin to Mekelle University Medical Microbiology Laboratory and were examined using the concentration method within 8 h after collection. After 72 h, Kato–Katz preparations were re-examined to detect helminth ova. A child was categorized as infected if the stool sample was positive for any parasite by any of the methods used. To ensure quality, each slide was examined twice by two of the three experienced laboratory technician independently.

### Quality control

To control our data quality, 10% of the total positive specimens were randomly selected and re-examined by three experienced laboratory technicians who did not have any information about the previous results. As results among the laboratory technicians were similar, the results of the new laboratory examinations were therefore used as quality control.

### Data analysis

Data were analysed with Stata Release 15. Confidence intervals for prevalence were calculated using the Agresti-Coull formula. Negative binomial regression was used to model prevalence rate ratios. Prevalence rate ratios have several advantages over odds ratios. The first is that they are simple to interpret; they directly compare prevalence, so a prevalence ratio of 2 means that prevalence is twice as high. Second, prevalence ratios, but not odds ratios, have a mathematical property called collapsibility; this means that the size of the risk ratio will not change if adjustment is made for a variable that is not a confounder [28]. All reported *p*-values were two-tailed, and statistical values were considered significant when *p* < 0.05.

## Results

### Characteristics of participants in the study

Most mothers (79%) had one under-five child. According to the results of the study, 58% mothers and 44.9% of the fathers were illiterate (unable to read or write). As to the shoe wearing habit of the children, majority, 587 (96.2%) of them wore shoes either sometimes or regularly. Only 32(5.2%) of the children consumed raw vegetables at a regular basis. Regarding the child deworming status, 395 (68.3%) of the children were dewormed for STH. Household water treatment (any means) was practiced among 150 (24.6%) households. Regarding latrine availability, 59.7% of the households had their own latrine facilities (Table 1).

**Table 1:** Prevalence of IPIs and Anaemia among 610 pre-school children in Tigray, Ethiopia, 2017

Risk factors Categories		No (%)(*n* = 610)	IPI Prevalence %	Anaemia Prevalence %
Sex	Girls	284 (46.6)	57.4	19.7
	Boys	326 (53.4)	58.6	23.3
Age (months)	6–11	32 (5.3)	46.9	28.1
	12–23	108 (17.7)	50.0	43.5
	24–35	160 (26.2)	54.4	26.9
	36–47	149 (24)	61.1	14.8
	48–59	161 (26.4)	66.5	6.8
The habit of a child playing in soil	Never	23 (3.8)	52.2	30.4
	Sometimes	135 (22.1)	54.1	25.9
	Regularly	452 (74.1)	59.5	19.9
Child shoe wearing habit	Never	49 (8)	59.2	22.4
	Sometimes	202 (33.1)	56.4	20.3
	Regularly	359 (58.9)	58.8	22.3
Family monthly income in Ethiopian birr	< 500 birr	99 (16.2)	62.6	25.3
	500-2000birr	451 (73.8)	57.6	22.4
	> 2000 birr	60 (9.8)	53.3	10.0
Consumption of raw vegetables	Never	106 (17.4)	58.5	17.0
	Sometimes	472 (77.4)	57.6	22.5
	Always	32 (5.2)	62.5	25.0
Child fingernail status	Untrimmed	295 (48.4)	59.3	19.3
	Trimmed	315 (51.6)	56.8	23.8
Child hand cleanness	Unclean	243 (39.8)	63.4	16.5
	Clean	367 (60.2)	54.5	25.1
Child hand washing habit with soap before a meal	Never	80 (13.1)	60.0	23.8
	Sometimes	344 (56.4)	57.3	20.9
	Always	186 (30.5)	58.6	22.0
Number of children in the house	< 4	144 (23.6)	59.4	22.5
	≥4	466 (76.4)	53.5	18.8
Number of under five children in the house	1	482 (79)	61.0	20.7
	2 and more	128 (21)	46.9	25.0
Mother’s educational level	Illiterate	354 (58)	61.6	24.0
	Primary school	193 (31.6)	53.9	17.6
	Secondary school	54 (8.9)	50.8	20.6
Mother’s/guardian’s Occupation	House wife	562 (92.1)	58.2	22.2
	Governmental employee	19 (3.1)	42.1	15.8
	Self-employed	29 (4.8)	65.5	13.8
Household water source	Unprotected	470 (77)	58.9	20.9
	Protected	140 (23)	55.0	24.3
Solid waste disposal	Improper	559 (91.7)	62.7	15.7
	Proper	51 (8.3)	57.6	22.2
Use household water treatment	No	460 (74.4)	55.7	23.3
	Yes	150 (24.6)	65.3	16.7
Toilet availability	No	246 (40.3)	58.1	18.7
	Yes	364 (59.7)	58.0	23.6
Child contact with pet animals	Never	366 (60)	56.0	24.0
	Sometimes	149 (24.4)	62.4	19.5
	Regularly	95 (15.6)	58.9	15.8
Child deworming (*n* = 578)	No	183 (31.7)	49.7	27.3
	Yes	395 (68.3)	61.6	19.2
Diarrhea in the last 14 days	No	437 (71.6)	53.8	21.5
	Yes	173 (28)	68.8	22.0
Current GI symptoms	No	475 (77.9)	53.9	21.5
	Yes	135 (22)	72.6	22.2

### Prevalence of intestinal Parasitosis and anaemia

Among the 610 participating pre-school children, 354 (58%) (95% CI: 54.1–62.1%) were infected by one or more parasitic organism. Single, double, and triple parasitic infections were seen in 249 (40.8%), 83 (13.6%), and 22 (3.6%) children, respectively. Seven different intestinal parasitic organisms were detected with *E. histolytica/dispar* the most prevalent 220 (36.1%) followed by *G. lamblia* 128 (20.1%) and *H. nana* 102 (16.7%). Mixed infections were common among children positive for *G. lamblia, E. histolytica/dispar* and *Cryptosporidium* spp. Soil transmitted helminthic infections either alone or with other intestinal parasite were identified in 104 (17.1%) of the children with *H .nana* 102 (16.7%) being dominant while *Ascaris*, hookworm and *Trichuris* infections were very rare or absent. The prevalence of any type of anaemia was 21.6% (95% CI: 18.5–25.1%) (Table 2).

**Table 2:** Intestinal parasite and anaemia among 610 pre-school children in the Tigray region of Northern Ethiopia, 2017

Variables	No (%)
Children with intestinal parasite(s) detected	354 (58)
Number of single parasitic infections	249 (40.8)
Number of double parasitic infections	83 (13.6)
Number of triple parasitic infections	22 (3.6)
Haemoglobin level	
Normal	478 (78.4)
Anaemia	132 (21.6)
Infection type	
Protozoa	
*E. histolytica*/dispar	125 (20.5)
*G. lamblia*	52 (8.5)
*Cryptosporidium* spp.	11 (1.8)
*E. vermicularis*	2 (0.3)
Helminths	
*H. nana*	59 (9.7)
*A. lumbricoides*	1 (0.2)
*S. stercoralis*	1 (.2)
Double infection	
*G. lamblia + E. histolytica/dispar*	44 (7.2)
*G. lamblia + H. nana*	8 (1.3)
*E. histolytica/dispar + H. nana*	27 (4.7)
*G. lamblia + Cryptosporidium* spp. Oocyst	2 (0.3)
*E. histolytica/dispar + E. vermicularis*	2 (0.3)
Triple infection	
*G. lamblia + E. histolytica/dispar + H. nana*	8 (1.3)
*E. histolytica/dispar + G. lamblia + Cryptosporidium* spp.	14 (2.3)

### Socio-demographic and health related factors associated with IPIs and Anaemia

Prevalence of intestinal parasitic infection rose from 50% in children aged under 2 years, to 66% in children aged 4 to 5 years. The prevalence rate increased by 7.8% per year over the age range studied (negative binomial regression, incidence rate ratios (IRR) =1.078, *p* = 0.009). In contrast to IPIs, prevalence of anaemia rose from 28% in those aged 6 months to 1 year to 44% in children aged 1 year. Thereafter it declined sharply with age to reach 7% in those aged 4 years (Table 1).

Because prevalence of parasites is associated with age, we used negative binomial regression adjusted for age to calculate prevalence rate ratios associated with each of the associated risk factors. Adjusted for age, prevalence of IPI was higher in children with current gastro intestinal (GI) symptoms (*p* = 0.001), who had diarrhea in the previous 14 days (*p* = 0.000), and who had been de-wormed (*p* = 0.045). Prevalence was however, lower in households with two or more children aged under 5 (*p* = 0.015) (Table 3).

**Table 3:** Associated Risk factors for prevalence of IPIs among 610 pre-school children in the Tigray, Ethiopia, 2017

Risk factor	Prevalence ratio	95% CI	*p* value
Socio-demographic characteristics			
Male sex	1.0	0.89**–**1.20	0.714
2+ under five children in the house	0.76	0.61–0.95	0.015**
Any education	0.89	0.74**–**1.10	0.186
4 or more children	0.93	0.72**–**1.20	0.611
Hygienic and environmental characteristics			
Child’s hands clean	0.89	1.0**–**0.80	0.129
Solid waste disposed of properly	0.91	0.76**–**1.10	0.327
Water from improved source	1.0	0.85**–**1.10	0.382
House hold water treatment	1.2	1.0**–**1.40	0.057
Presence of domestic animals in house	1.0	0.78**–**1.30	0.939
Soap used in handwashing	1.0	0.89**–**1.10	0.988
Habit of eating raw meat	1.0	0.75**–**1.40	0.855
Latrine availability in the house	1.0	0.92**–**1.10	0.949
Child’s fingernails trimmed?	1.0	0.89**–**1.10	0.588
Eats fruit or raw vegetables	1.0	0.82**–**1.20	0.798
Health characteristics			
Child in contact with animals	1.1	1.0**–**1.20	0.230
Any skin disease	1.1	0.95**–**1.20	0.258
Did the child get dewormed(*N* = 427)	1.2	1.0–1.40	0.045**
Diarrhea in the last 14 days	1.3	1.2–1.40	0.000**
Current GI symptoms	1.3	1.1–1.60	0.001**

** = statistically significant. All prevalence ratios are adjusted for age using negative binomial regression

### Factors associated with anaemia among pre-school age children

Eating raw meat (p = 0.000), any maternal education (p = 0.000), and household water treatment (*p* = 0.044), were associated with lower prevalence ratio of anaemia, when adjusted for age. On the other hand, presence of domestic animals in the house (*p* = 0.006), and proper disposal of solid waste (*p* = 0.009) were both associated with increased risk (Table 4). There was a 25% prevalence of anaemia in the 256 children who had no intestinal parasites, compared with a 19% prevalence in the 354 who had any parasite. This association was not statistically significant (*p* = 0.085, Chi-squared test). Adjusting prevalence for site and child’s age did not substantively alter this finding.

**Table 4:** Risk factors for anaemia among 610 pre-school children in the Tigray, Ethiopia, 2017

Risk factors	Prevalence ratio	95% CI	P- value
Habit of raw meat eating of the child	0.49	0.45–0.54	0.000**
4 or more children	0.64	0.39–1.00	0.075
Any maternal education	0.64	0.52–0.79	0.000**
House hold water treatment	0.75	0.56–1.00	0.044**
Soap used in handwashing	0.85	0.64**–**1.10	0.279
Child in contact with animals	0.87	0.6**–**1.20	0.399
Any skin disease	0.95	0.56**–**1.60	0.839
Diarrhea in the last 14 days	1.0	0.66**–**1.40	0.832
Current GI symptoms	1.0	0.59**–**1.60	0.898
Did the child get dewormed (N = 427)	1.0	0.86**–**1.20	0.891
Water from improved source	1.0	0.82**–**1.30	0.752
Child’s fingernails trimmed	1.1	1.0**–**1.20	0.077
Male sex	1.1	1.0**–**1.30	0.134
Child’s hands clean	1.2	0.87**–**1.60	0.280
Latrine availability in the house	1.2	0.84**–**1.70	0.308
Number of under-five children in the house	1.3	0.92**–**1.90	0.129
Eats fruit or veg raw	1.4	0.91**–**2.00	0.133
Presence of domestic animals in house	1.5	1.1–2.00	0.006**
Solid waste disposed of properly	1.5	1.1–2.10	0.009**

** Statistically significant. All prevalence ratios are adjusted for age using negative binomial regression

## Discussion

IPI prevalence (58%) in our study was comparable with previous reports from Pakistan 52.8% [29] and Nigeria, 51.4% [30], though lower than studies conducted in south Ethiopia, 85.1% [20], Cuba, 71.1% [31] and Malaysia, 76.5% [32]. However, our prevalence is higher than previous similar studies conducted in east Ethiopia, 24.3% [22], south Ethiopia 4.9% [34], Amhara region, 15.5% [33], Oromia region, 49.6% [34], Shoa, Ethiopia 17.4% [35] and Northwest Ethiopia 25.8% [36]. Others have reported lower IPI prevalence in Nigeria, 13.7% [37], Iran, 26.6% [38]**,** Uganda, 26.5% [39], Saudi Arabia, 17.7% [40] and Yemen, 30.9% [41]. These reported differences in IPI prevalence might be due to the difference in parasitological methods used, geographical location, level of environmental sanitation, drinking water source, season, family education, personal- hygiene, parental socioeconomic and cultural difference of the study participants.

One explanation for the high IPI prevalence in this study could be because of seasonal variation. Data collection for our study took place during the rainy season in Ethiopia, other studies [32, 35, 40, 42] were conducted in the dry season. Seasonal variation may be explained by increased contamination of water sources (e.g., rivers, streams, and wells) with human excreta from open defecation which is the main risk factor for diarrhoeal disease and IPIs, especially children who routinely play in the an unhygienic environment [25]. In addition, as 77% of households in our study used unprotected water sources, the main factor for faecal-oral transmitted disease such as IPIs, this may also contribute to the high IPI prevalence.

Another possible reason for the high prevalence of IPIs in our study is the laboratory methods we employed. We used the modified Ziehl Nelson method to detect *Cryptosporidium* spp., whereas the other studies except [38, 43] did not. Furthermore, some studies isolated only the soil-transmitted helminths (STH) and not protozoa which would artificially decrease IPI prevalence [22, 32, 33, 39].

*E. histolytica, G. lambilia* and *C. parvum* were the most prevalent protozoan parasites in our study cohort. The high prevalence of *E. histolytica/dispar* (36.1%) in our study is in agreement with previous reports [44]. Consequences of childhood *E. histolytica* infection include malnourishment, anaemia and stunted growth [45]. However, others have reported *G. lamblia* [41, 46] and *C. parvum* [47] as the dominant parasites. The difference in prevalence of enteric protozoa may be due to differences in contamination of drinking water sources, availability of toilets, handwashing and consumption of raw vegetables of the study participants.

Helminthic intestinal parasites, particularly soil transmitted helminths (STHs), commonly infect children in low to middle-income countries. In our study, helminthic infections were identified in 104 (17.1%) of the children with *H .nana* 102 (16.7%) being dominant while *Ascaris*, hookworm and *Trichuris* infections were very rare or absent. The low STH incidence in our study might be due to the initiation of a national deworming program, as the majority, 68.3% of the children were dewormed during the data collection time and deworming is given on a regular basis. The relatively high prevalence of wearing shoes either regularly or sometimes and low prevalence of consumption of raw vegetables may also partially explain the low STH prevalence. Besides, the prevalence of STH might also be due to differences in environmental factors such as climate, topography [48], surface temperature, altitude, soil type and rainfall [49].

Our study shows that dewormed children were more infected with IPIs than their counter part which appears counter-intuitive. This could be due to the fact that the prevalence of STHs, where deworming is recommended, was very small in our study. Whereas, protozoan infection where deworming is not given was the most dominant. A lower prevalence of STH compared with *H. nana* has been previously reported in Peru [50] and in Eastern Ethiopia in elementary school children receiving regular albendazole deworming treatment [51]. This could be probably due to the fact that albendazole has little effect on *H. nana* unlike *Ascaris lumbricoides*, *Trichuris trichiura* and hookworm [52].

Children aged 48–59 months (PR = 1.078, *p* = 0.009) were more likely to be infected by IPIs compared with younger children. This likely reflects increased risk of IPI exposure from playing activity of the older, more mobile children within unhygienic external environments and hence exposed to faecal-contaminated soil.

IPIs were significantly higher in children with current gastro intestinal (GI) symptoms and in those who had diarrhea in the previous 14 days. This was similar to the report from Iran [53]. This is due to the fact that most of the isolated IPIs in this study, *E. histolytica* and *G.lamblia,* are potential causes of diarrhea. Likewise, STHs are usually accompanied by current gastro intestinal (GI) symptoms.

Only a quarter of households used household water treatments, mainly chlorination and boiling. Though not statistically significant, children whose family did not treat their water were 1.2 times (*p* = 0.057) more likely to be infected by IPIs. Chlorination is less expensive, less time consuming and provides residual disinfection against recontamination and significantly reduces diarrhea [54]. However, due to the smell and taste [55], only a few families use chlorination in our study. Similarly, due to the consumption of fuel, time, and lack of residual protection against re-contamination [56], only a few households use boiling. Hence a cheap, point of use technology, such as solar disinfection (SODIS), which overcomes these limitation is required by such communities to tackle the consumption of unsafe drinking water.

The prevalence of childhood anaemia among pre-school children in our study was 21.6%, which is comparable with studies from south central Ethiopia, 28.2% [57], Kenya, 25% [58], Malaysia, 26.2% [32], and Brazil 28.9% [59]. Our study was, however, lower than other reports from Ethiopia, 37.3–42.2% [60, 61], the 2016 Ethiopian Health Demographic Survey, 57% [19], the EDHS anaemia prevalence of the Tigray regional state, 54% (19) and the WHO classification for anaemia, 40% [13].

Likewise, studies performed outside Ethiopia have reported higher anaemia prevalence including those conducted in Brazil, 32.8% [62], Uganda, 37.2% [14], Nigeria, 38.6% [63], Bangladesh, 51.9% [64], Senegal,53.4% [65], West Africa, 51.8% [66], Tanzania, 47.6% [67] and Brazil, 45.1% [62].

Possible reasons for the variation in anaemia prevalence could be due to differences in maternal education [57] concomitant childhood malaria superinfection [55], family income [58], drinking water source [51], personal-hygiene [68] and STH infections [54, 57]. For example, the high anaemia rate in the studies such as Tanzania, Kenya, Senegal, Uganda, south Ethiopia, was due to concomitant malaria infection. Seasonality may have also affected the anaemia prevalence in our study as malaria prevalence is usually low during the rainy season [69] where our data collection time was from June–August, the rainy season of Ethiopia. In addition, our study participants were from the community rather than from health institutions, and the prevalence of STH, the main cause of anaemia [58, 70] was very low in our study.

In this study, children whose mothers were not educated were significantly anaemic compared their counter parts. This was similar with the study conducted in Tanzania [71]. This protective benefit of maternal education could be related to an increased knowledge for adequate healthcare and nutrition for children hence its possibility for decreasing the risk of anaemia. Similarly children who eat meat were less anaemic compared to those who don’t eat meat. This was supported by a study from Israel [72]. This is due to the fact that meat is a good source of Iron; hence without enough iron, less hemoglobin and fewer red blood cells are made, leading to anaemia. It should be also noted that anaemia has a variety of causes though 50% of all cases is caused by iron deficiency [53]. In this study, anaemia was negatively associated with household water treatment of mothers. This is because intestinal helminths and diarrhoea, which cause anaemia [16], can be killed with household water treatments methods [5, 6, 54].

### Limitation of the study

One of the limitations of our study was that differentiation between the morphologically identical species of Entamoeba was not within the scope of this study, as only conventional microscopy was used to detect the amoebae.

## Conclusions

This study revealed the significant burden of IPI and anaemia in preschool children in a rural community in Northern Ethiopia. More than half of the children were infected with intestinal parasites and one in five were anaemic. IPI was higher in children with current gastro intestinal symptoms, who had diarrhoea in the previous 14 days, and who had been de-wormed. Prevalence was however, lower in households with two or more children aged under 5 years. To address the problem of childhood IPIs, efforts are required by designing and implementing prevention strategies, such as integrating mothers’/guardian’s education on personal and environmental hygiene into existing national health extension program.

Eating raw meat, any maternal education and any household water treatment were associated with lower prevalence ratio of anaemia. On the other hand, presence of domestic animals in the house and proper disposal of solid waste were both associated with increased risk. This study underlines the need for interventions focusing on the identified modifiable risk factors to prevent long-term morbidity and give these children the maximal opportunity for health in the future.

Hb Haemoglobin.

## Data Availability

The datasets used and analyzed during the current study are available from the corresponding author on reasonable request.

## Abbreviations

IPIs: Intestinal parasitic infections
EDHS: Demographic and Health Survey of Ethiopia
WHO: World Health Organization
MZN: Modified Ziehl-Neelsen
